# Physiological effects of high‐altitude trekking on gonadal, thyroid hormones and macrophage migration inhibitory factor (MIF) responses in young lowlander women

**DOI:** 10.14814/phy2.13400

**Published:** 2017-10-25

**Authors:** Vittore Verratti, Francesca Ietta, Luana Paulesu, Roberta Romagnoli, Ilaria Ceccarelli, Christian Doria, Giorgio Fanò Illic, Camillo Di Giulio, Anna M. Aloisi

**Affiliations:** ^1^ Department of Neuroscience, Imaging and Clinical Sciences “G. d'Annunzio” University of Chieti‐Pescara Chieti Italy; ^2^ Department of Life Sciences University of Siena Siena Italy; ^3^ Department of Medicine, Surgery and Neuroscience University of Siena Siena Italy; ^4^ Libera Università di Alcatraz Umbria Italy

**Keywords:** Hormones, macrophage migration inhibitory factor, women at high altitude

## Abstract

Altitude hypoxia is often associated with impairment of human reproduction. In this study, hormones and macrophage migration inhibitory factor (MIF, a proinflammatory cytokine with key roles in human reproduction) were determined in seven regularly menstruating, lowlander native women living at sea level participating in 14 days of trekking at moderate and high altitude. Blood and saliva samples were collected from each subject at high altitude (5050 m a.s.l. [above sea level]), and at sea level before and after the expedition. Testosterone level was lowered by high altitude and was restored after the end of the expedition, while progesterone decreased significantly in all participants at the end of the expedition, although most of the participants were in the luteal phase. The salivary concentration of MIF decreased greatly at altitude, but its levels were completely restored after the return to sea level. Our findings showed high sensitivity and rapid changes in the determined parameters in response to the high‐altitude hypoxic environment, particularly MIF.

## Introduction

Blood oxygen levels are crucial for survival as oxygen serves basic cell functions and complex mechanisms in all physiological systems. However, some environmental conditions, for example, high altitude, can significantly affect oxygen availability, with consequent short‐ and/or long‐term body adjustments. About that, it is well known that chronic exposure to altitude causes reversible fertility impairments, in humans who are not well adapted (Donayre et al. 1968; Okumura et al. 2003; Verratti et al. 2007, 2016a; Pelliccione et al. 2011), but it does not cause adverse effects on fertility in populations that settle permanently in regions of high altitude (Farias et al. 2005).

Regarding the female reproductive system, studies on high‐altitude residents have shown that pregnancy and neonatal conditions can be adversely affected by “high altitude.” Intrauterine growth restriction reduces birth weight in high‐altitude infants (Jensen and Moore 1997). Pre‐eclampsia is more common in mothers living at high altitude than low altitude (Harrison and Moore 1990; Zamudio et al. 1993, 1995). Moreover, studies on the influence of the menstrual cycle phase on acclimatization to high altitude have dealt with sympathetic nerve activity and other various metabolic activities (Mazzeo et al. 1998; Brutsaert et al. 2002). Compared to the lowlander native women living at sea level, high‐altitude native women living at high altitude have a shorter reproductive life span because of delayed menarche and earlier menopause (Gonzales and Villena 1996), a longer duration of the follicular phase and an earlier occurrence of ovulation after the peak of luteinizing hormone (LH) (Escudero et al. 1996). On the other hand, an extensive study of indigenous Andean population showed no significant changes in the menstrual cycle (length or regularity) compared to lowlander native women living at sea level (Vitzthum 2001). The high‐altitude indigenous habitants (Himalayan and Andean) seem to have adapted to the hypoxic environment without clear and notable negative impacts on fertility (Vitzthum and Wiley 2003; Vitzthum 2013).

Gonadal hormones are the key factors in reproductive capacity, but also in the neural control of respiration. Testosterone, estradiol, and progesterone were found to be affected by hypoxia, while sex hormones are known to cross the blood–brain barrier, and their receptors have been found in central and peripheral structures that regulate respiration (Behan and Kinkead 2001). Progesterone levels detected during the luteal phase of high‐altitude native women inhabitants living at high altitude were found to be lower than those in comparable samples of lowlander native women living at sea level (Escudero et al. 1996; Jiang et al. 2000; Vitzthum 2001).

Another important molecule related to reproduction is macrophage migration inhibitory factor (MIF), occurring in the cycling uterus in preparation for pregnancy (Paulesu et al. 2010). MIF is also produced at the implantation site by the human placenta, and MIF production is regulated by the low oxygen tension, characterizing the uterine environment in early gestation (Ietta et al. 2007). The importance of MIF during the establishment of pregnancy was revealed by Yamada et al. (2003), who showed that women suffering recurrent early miscarriages had lower maternal MIF plasma levels.

The aim of this study was to examine the hormone and MIF responses in seven healthy, regularly menstruating, native women living at sea level who participated in the Gokyo Khumbu/Ama Dablam Trek 2012‐Scientific Expedition, involving 14 days of trekking at moderate and high altitude.

## Materials and Methods

The Gokyo Khumbu/Ama Dablam Trek 2012 was a broad research project involving different areas of physiology, as evidenced by recent publications (Pietrangelo et al. 2015; Mancinelli et al. 2016; Morabito et al. 2016; Scordella et al. 2016; Tam et al. 2016; Verratti et al. 2016b).

## Subjects

This study was carried out on seven fertile females (36.3 ± 7.1 years old) resident at sea level who reported a very regular menstrual cycle (28 ± 1 days per cycle). The voluntary participants were all healthy, with no history of pregnancy or chronic diseases. They were all moderately trained trekkers; none of them took steroid hormones before or during the trek. Each subject signed a “declaration of risk acceptance and assumption of responsibility,” and authorized personal data collection and processing. The study was approved by the Bioethics Committee of “G. d'Annunzio” University of Chieti‐Pescara (Italy, protocol no. 773 COET), and was designed in accordance with the recommendations of the Declaration of Helsinki.

### Experimental protocol

**Figure 1:**
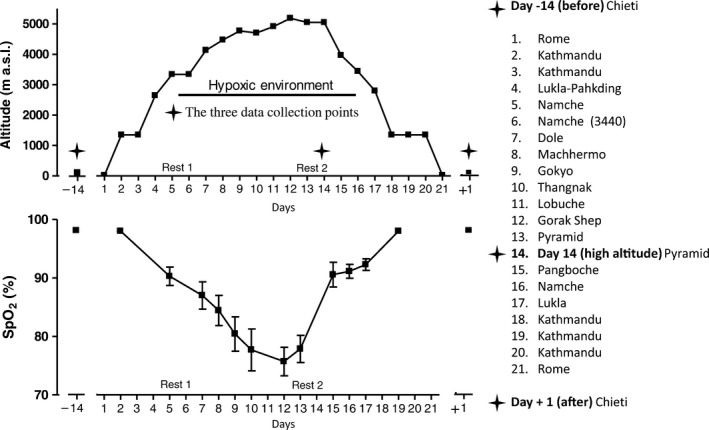
Upper: altimetry during the scientific expedition from Rome (day 1) to Rome (day 21) and at the data collection in Chieti before (day −14) and after (day +1) the expedition. Two days were spent at altitude, the first at Namche Bazaar (3400 m a.s.l.) and the second at Desio's Pyramid (5050 m a.s.l.). Lower: SpO_2_ (%) values recorded at different altitudes during the expedition and at sea level before (day −14) and after (day +1) the expedition. a.s.l., above sea level; SpO_2_, saturation of peripheral oxygen.

Blood (for hormone determinations) and saliva (for MIF determination) samples were collected three times: (1) at sea level before (day −14 [before]), (2) during the high‐altitude trekking phase (day 14 [high altitude]) and (3) at sea level, after high‐altitude trekking (Day+1 [after]), while the peripheral oxygen saturation (SpO_2_) measurements were made 14 times (including the three measurements carried out at day −14 [before], at day 14 [at high altitude], and at day+1 [after]), to better trace the saturation profile during shipment. The details of the three phases of the experimental protocol are given in Table 1. The altimetry map for the expedition is shown in Figure 1 (Upper). At each of the three data collection points, the volunteers were interviewed, menstrual cycle data were recorded, and blood and saliva samples were collected. The volunteers were interviewed about health conditions during the altitude trek.

**Table 1:** Details of the experimental protocol

1	Meeting at University of Chieti‐Pescara (110 m a.s.l., “Sea level before”) 14 days before the beginning of the expedition (day −14)
2	The Gokyo Khumbu/Ama Dablam Trek 2012‐Scientific Expedition started in Rome on 23 October 2012 (day 1) and ended in the same city on 12 November 2012 (day 21). After the flight from Rome to Kathmandu (Nepal), the expedition group remained in Kathmandu the following day to define organizational, managerial, and strategic activities for the high‐altitude trek. At day 4, the group flew from Kathmandu to Lukla (2800 m a.s.l.) where the altitude trek started. After 2 days of moderate‐altitude trekking the group reached Namche Bazaar (3440 m a.s.l.) where it spent 1 day at rest. The group then reached the Ev‐K2 Italian CNR Desio's Pyramid (5050 m a.s.l.) and rested there for 1 day (*High altitude*, day 14). The end of the trek at Lukla, on day 17, coincided with the end of the hypoxic experience for the expedition members. The group then flew to Kathmandu and then back to Rome (day 21). In total, the scientific expedition lasted 21 days with 14 days of trekking at moderate and high altitude including 2 days spent at rest at altitude.
3	Meeting at University of Chieti‐Pescara (110 m a.s.l., “Sea level after”) 1 day after the end of the expedition (day +1).

a.s.l., above sea level.

### Measurements

#### Saturation of peripheral oxygen

SpO_2_ was recorded for about 2 min by pulse oximetry from the fingertip (503 OXY‐5 GIMA; Gima S.p.A., Gessate, Italy). SpO_2_ parameter specifications: measuring range (35–99%); accuracy (±2% during 75–99%, ±3% during 50–74%).

#### Hormones

Women of reproductive age undergo hormonal changes during the menstrual cycle. However, we chose not to artificially alter the hormonal cycles and not to synchronize the cycle in the subjects, in order to examine the potential impact of altitude on menstrual cycle physiology. Therefore, the subjects were in different menstrual phases at the time of the sampling.

Blood samples (5 mL) were collected by venipuncture in the morning, and centrifuged (3000*g* for 10 min), after which serum samples were frozen in liquid nitrogen. On the day of testing, they were thawed and assayed for the following hormones: thyroid‐stimulating hormone (TSH), LH, follicle‐stimulating hormone (FSH), prolactin, testosterone, estradiol, progesterone, free triiodothyronine (FT3), free thyroxine (FT4), and cortisol (ADVIA Centaur^®^ CP Immunoassay System; Siemens Healthcare, Milan, Italy).

#### Salivary MIF

A saliva sample was taken in the morning, right after the blood sample, by Salivette^®^ Cotton Swab (Starstedt, Nümbrecht, Germany). Participants were asked to gently chew the swab for approximately 2 min. Following each collection, the swab was frozen at −20°C until taken to the laboratory at University of Siena (Italy). On the day of sample analysis, the samples were centrifuged after thawing and assayed for the cytokine MIF using a colorimetric sandwich enzyme‐linked immunosorbent assay protocol developed by Ietta et al. (2002) at the University of Siena, Italy. Intra‐ and interassay coefficients of variation were 3.86 (0.95) and 9.14 (0.47), respectively. The MIF concentration was expressed as ng/mL of saliva.

### Statistical analysis

All collected data were analyzed by Friedman ANOVA for the three data collection points (Day −14 [before], Day 14 [high altitude], and Day +1 [after]), followed by Wilcoxon matched pairs test when appropriate to evaluate the effect of altitude on the different parameters (details are reported in the Results section). In the figures and tables, the data are expressed as mean ± SEM.

## Results

The expedition was successful with no serious organizational problems; weather conditions were good, with dry sunny days and with an average temperature of about 11°C at Lukla (2800 m a.s.l.), 7°C in Namche Bazar (3440 m a.s.l.), and −1°C at Gorak Shep (5186 m a.s.l.), as recorded throughout October and November. No significant health problems and no alterations of menstrual cycle length were recorded during the altitude trek in any of the subjects. Table 2 shows the changes in weight for each subject by altitude.

**Table 2:** Body weight of participants in the high‐altitude expedition

Subject ID	Age	Body weight (kg)		
		Sea level before	High altitude	Sea level after
1	41	68.6	70.5	67
2	32	56.5	56.4	55.8
3	28	60.4	58.4	58.8
4	48	60.7	64.1	62
5	31	59	60	60
6	36	49.8	49.1	49
7	37	85	84.3	84.5

### Saturation of peripheral oxygen

As expected, SpO_2_ was significantly affected by altitude (Friedman ANOVA [*N* = 7, df=2] = 11.76, *P* < 0.002). There was a progressive decrease in oxygenation until day 12, followed by a return to normal values at the end of the expedition (Fig. 1, Lower).

### Hormones

#### Estradiol

**Figure 2:**
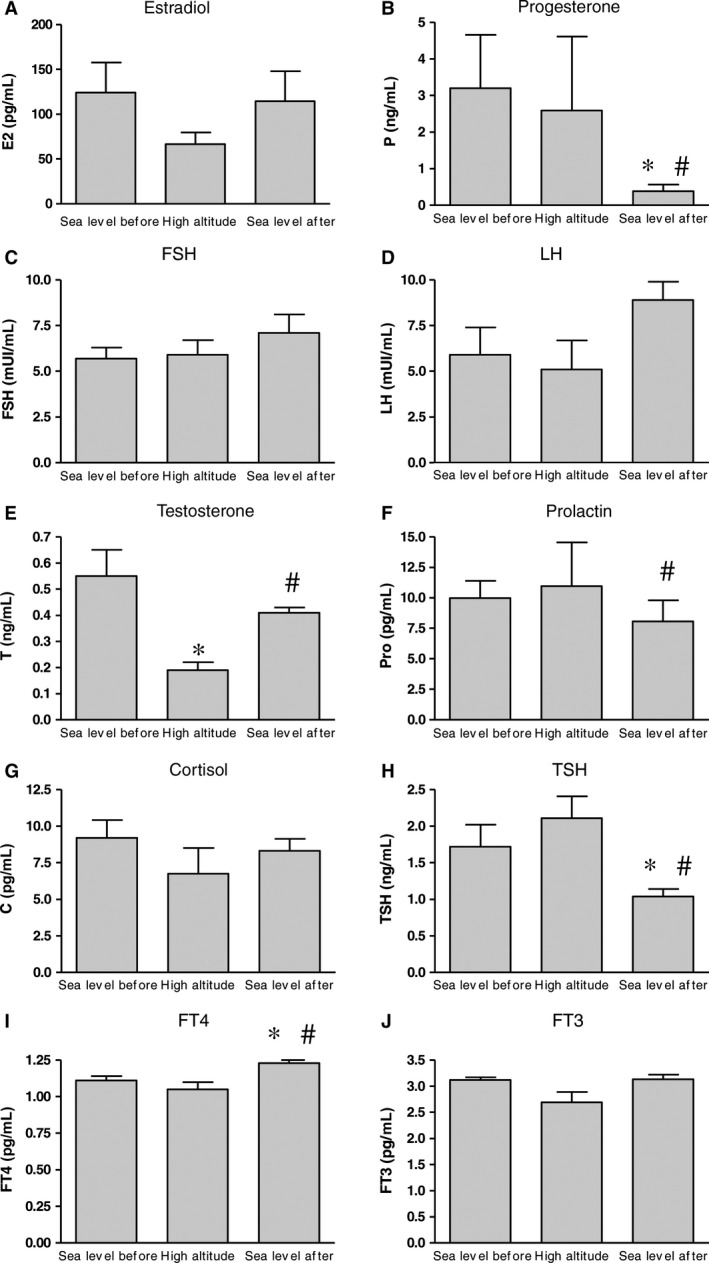
(A‐J) Serum hormone levels at the three data collection points (sea level before‐high altitude‐sea level after). *P < 0.05 versus sea level before, ^#^ *P* < 0.05 versus high altitude. (A) average estradiol (E2) value (pg/mL); (B) average progesterone (P) value (ng/mL); (C) average follicle‐stimulating hormone (FSH) value (mUL/mL); (D) average luteinizing hormone (LH) value (mUL/mL); (E) average testosterone (T) value (ng/mL); (F) average prolactin (Pro) value (pg/mL); (G) average cortisol (C) value (pg/mL); (H) average thyroid‐stimulating hormone (TSH) value (ng/mL); (I) average free thyroxine (FT4) value (pg/mL) and (J) average free triiodothyronine (FT3) value (pg/mL) among the three determinations.

There were no significant differences in estradiol levels among the three determinations (Friedman ANOVA [*N* = 7, df=2] = 2, *P* = 0.36), although, as seen in Figure 2A, the values tended to decrease at high altitude compared to sea level (recorded before and after the expedition).

#### Progesterone

Progesterone levels (Fig. 2B) showed significant changes among the data collection points (Friedman ANOVA [*N* = 7, df=2] = 9.5 *P* < 0.008) with lower values at sea level after the expedition than during the first two points (*P* < 0.04).

#### FSH and LH

FSH and LH levels (Fig. 2C and D, respectively) did not show significant differences among the three data collections (Friedman ANOVA [*N* = 7, df=2] = 3.71, *P* = 0.15 and [*N* = 7, df=2] = 2.00, *P* = 0.37, respectively).

#### Testosterone

Testosterone levels (Fig. 2E) differed significantly among the three data collection points (Friedman ANOVA [*N* = 7, df=2] = 12.07, *P* < 0.002), with lower levels recorded at altitude than at sea level before and after the expedition (*P* < 0.01 both).

#### Prolactin

Prolactin levels (Fig. 2F) differed significantly among the three data collection points (Friedman ANOVA [*N* = 7, df=2] = 6.00, *P* = 0.04), with lower values at sea level after the expedition than at altitude (*P* < 0.02).

#### Cortisol

Cortisol levels (Fig. 2G) did not show significant changes among the three data collection points (Friedman ANOVA [*N* = 7, df=2] = 0.28, *P* = 0.86).

#### TSH

TSH levels (Fig. 2H) differed significantly among the three data collection points (Friedman ANOVA [*N* = 7, df=2] = 9.55, *P* < 0.008), with lower levels at the third data collection point than at the other two (*P* < 0.01 and 0.02, respectively).

#### FT3 and FT4

FT3 levels did not show significant changes in any of the three data collection points (Friedman ANOVA [*N* = 7, df=2] = 3.71, *P* = 0.15, Fig. 2J), while FT4 (Fig. 2I) did differ significantly (Friedman ANOVA [*N* = 7, df=2] = *P* < 0.04), with higher levels at the third point than at the first and second (*P* < 0.03 and *P* < 0.02, respectively).

### Migration inhibitory factor

**Figure 3:**
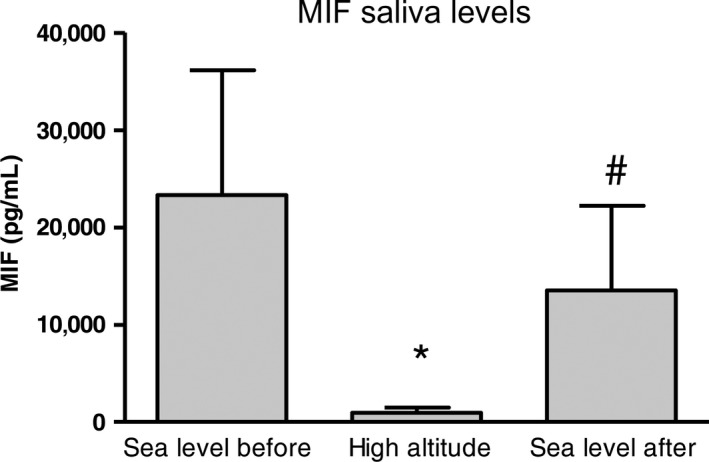
Salivary MIF concentrations at the three data collection points. *P < 0.05 versus sea level before; ^#^ *P* < 0.05 versus high altitude. MIF, migration inhibitory factor.

As shown in Figure 3, Friedman ANOVA applied to salivary MIF revealed significant differences among the three data collection points ([*N* = 6, df 2] = 9.00, *P* < 0.01), highly decreased lower levels observed in samples collected at high altitude compared to sea level (*P* < 0.02, both points).

## Discussion

The young women participating in the Gokyo Khumbu/Ama Dablam Trek 2012‐Scientific Expedition (14 days of trekking at moderate and high altitude) were subjected to high‐altitude hypoxia, resulting in a significant decrease in plasma testosterone and salivary MIF, while, from statistical point of view, progesterone significantly decreased, after high‐altitude exposure, similarly to prolactin.

The results of this study, particularly those on progesterone, clearly suggest the possibility of long‐lasting changes in the hypothalamus–pituitary–gonadal axes, supporting the importance of hormonal studies, not only before and during but also after the return to the sea level. Indeed, in accordance with previous studies on native women living at sea level and temporarily exposed to altitude (Reeves et al. 2001), the progesterone levels determined at high altitude showed no significant difference from those obtained in the subjects in the same menstrual phase at sea level before the expedition. However, an important inhibitory effect on progesterone secretion became evident at the data collection carried out after the return to sea level when all women were in the luteal phase (18–28 days) of the menstrual cycle: the progesterone levels were expected to be over 5 ng/mL, whereas the recorded levels were close to zero, a value below any acceptable concentration limit. These results clearly show that no ovulation occurred in any woman during the expedition. Moreover, the increased FSH and LH levels after the end of the expedition (although not statistically significant) support an insufficient suppressive effect on the pituitary gland by progesterone.

The testosterone levels were also reduced by altitude, but this effect was limited to the high‐altitude exposure; hence, this reduction can be attributed to a direct effect of hypoxia. On the other hand, we demonstrated previously that testosterone plasma levels can be increased by O_2_ supplementation in young adult men in hyperbaric conditions (Passavanti et al. 2010).

Estradiol tended to decrease by altitude, as a possible direct consequence of the lack of testosterone as a substrate for aromatase activity (androgen to estrogen). Interestingly, Perez‐Sepulveda et al. (2015) reported lower aromatase expression in the placenta from pregnancies complicated by pre‐eclampsia (Zamudio et al. 1995). A lower 17‐*β* estradiol/testosterone ratio was also found in the maternal circulation of pregnant women suffering from pre‐eclampsia (Perez‐Sepulveda et al. 2015).

The ability of hypoxia to modulate the hypothalamic–pituitary–gland axis was carried out mostly in men. In these studies, TSH, the pituitary factor, and the thyroid hormones T3 and T4 were found to be differently affected in the different experimental conditions (Richalet et al. 2010). Thyroid hormones can play an important role in adaptation because, among the other effects, they change the functioning of erythrocytes, facilitating oxygen release to the tissues (Snyder and Reddy 1970). The slight increase in TSH, observed in this study at altitude (not significant) can be explained by the adaptation to the need to increase oxygen availability, the decrement observed at sea level after the trek can be explained by a feedback effect; indeed, the FT4 at that determination showed the highest value. Interestingly, prolactin changes were also similar to those observed for TSH with the lowest levels after the return to sea level. This result was expected, considering the common modulation of TRH not only on the TSH but also on prolactin production.

Another important aspect of this study was the inflammatory response to altitude shown by the MIF concentrations. We selected this cytokine based on previous studies of human reproduction and the proinflammatory role of MIF in different pathophysiological processes (Aloisi et al. 2005; Nishihira and Mitsuyama 2009; Paulesu et al. 2010).

In an important study of Aloisi and collaborators, on chronic pain patients, we found that in these subjects, MIF levels were very low and that its concentration was positively correlated with testosterone (Aloisi et al. 2005). In this study, we can hypothesize that similarly to patients, low testosterone condition (present in all women at high altitude) can reduce MIF availability. The low testosterone levels found in the trekkers in this study suggest a close relationship between these factors, which goes beyond their original roles in the control of reproduction. In spite of the extremely low levels in salivary MIF at high altitude, other studies showed that placental tissues from high‐altitude women inhabitants had higher MIF concentrations than those from lowland native women living at sea level (Ietta et al. 2007). We can hypothesize that in high‐altitude pregnant women, a compensative MIF response may occur locally, in the placenta to assure the maintenance of pregnancy. However, while this adaptation seems beneficial to fetal survival, it may become detrimental by contributing to the development of maternal pre‐eclampsia, a severe complication in pregnancy, more common in mothers living at high altitude than at low altitude (Zamudio et al. 1995; Cardaropoli et al. 2012).

Several other studies have shown adaptive biological mechanisms in high‐altitude pregnancies. Beall et al. (2004) observed that Tibetan women with genotypes for high oxygen saturation of hemoglobin had higher offspring survival than women with low oxygen saturation genotypes. In a more recent review paper, Illsley et al. (2010) clearly described how the insufficient oxygen in high‐altitude pregnancy leads to a decrease in fetal growth in favor of cell survival. The authors demonstrated that metabolic reprogramming in the placenta decreases mitochondrial oxygen consumption and increases anaerobic glucose consumption. Hence, oxygen supply is increased to assure fetal survival but at the cost of less glucose availability.

In this study, the cortisol levels were not significantly affected by altitude, unlike those of testosterone. However, as MIF is also part of the hypothalamus–pituitary–adrenal axis, it contributes to the response to stress (e.g., evidence shows that MIF is secreted by the same pituitary cells that secrete adrenocorticotropic hormone (ACTH), Bernhagen et al. 1993; Nishino et al. 1995; Petrovsky and Bucala 2000). The findings might also reflect an integrated systemic response to the various stimuli to which the subjects were exposed, including altitude, type of food, physical exhaustion, and others.

### Limitations of the study

This study has some limitations: (1) it involved a small number of participants (Donayre et al. 1968); (2) each participant was in her normal cycle. At the data collection points at sea level before the altitude expedition, three participants were in the follicular phase, two in the postovulatory and two in the luteal phase; (3) no controls were available for diet, climate, or time changes. All these limitations make the data preliminary, and any interpretation must be speculative. However, some points need to be emphasized in favor of the validity of the data: (1) our choice not to synchronize the cycle or to give oral contraceptives to the participants is advantageous for the study of menstrual cycle physiology; (2) all subjects were moderately but sufficiently trained to support the climb program, wisely planned in accordance with the load of exercise carried out and a correct altimetric adaptation.

## Conclusion

This study highlights the changes that lowlander native women living at sea level (temporarily exposed to high‐altitude zones) might undergo, probably due to the change in oxygen saturation during the ascent to high altitude, and suggests possible factors involved in reproductive impairment.

## Conflict of Interest

The authors declare that the research was conducted in the absence of any commercial or financial relationships that could be construed as a potential conflict of interest.
